# Mechanochromism and optical remodeling of multi-network elastomers containing anthracene dimers†
†Electronic supplementary information (ESI) available. See DOI: 10.1039/c9sc02580d


**DOI:** 10.1039/c9sc02580d

**Published:** 2019-07-26

**Authors:** Huan Zhang, Dezhi Zeng, Yifei Pan, Yinjun Chen, Yonghong Ruan, Yuanze Xu, Roman Boulatov, Costantino Creton, Wengui Weng

**Affiliations:** a Department of Chemistry , College of Chemistry and Chemical Engineering , Xiamen University , Xiamen , Fujian 361005 , P. R. China . Email: wgweng@xmu.edu.cn; b Department of Chemistry , University of Liverpool , Donnan Lab , G31, Crown Street , Liverpool , L69 7ZD GB , UK . Email: boulatov@liv.ac.uk; c Laboratoire Sciences et Ingénierie de la Matière Molle , ESPCI Paris , PSL University , Sorbonne Université , CNRS , F-75005 Paris , France . Email: costantino.creton@espci.fr

## Abstract

Multi-network elastomers are both stiff and tough by virtue of containing a pre-stretched stiff network that can rupture and dissipate energy under load. However, the rupture of this sacrificial network in all described covalent multi-network elastomers is irreversible. Herein, we describe the first example of multi-network elastomers with a reformable sacrificial network containing mechanochemically sensitive anthracene-dimer cross-links. These cross-links also make our elastomers mechanochromic, with coloration that is both persistent and reversible, because the fluorogenic moiety (anthracene dimer) is regenerated upon irradiation of the material. In proof-of-concept experiments we demonstrate the utility of incorporating anthracene dimers in the backbone of the sacrificial network for monitoring mechanochemical remodeling of multi-network elastomers under cycling mechanical load. Stretching or compressing these elastomers makes them fluorescent and irradiating them eliminates the fluorescence by regenerating anthracene dimers. Reformable mechanochromic cross-links, exemplified by anthracene dimers, hold potential for enabling detailed studies of the molecular origin of the unique mechanical properties of multi-network elastomers.

## Abstract 2

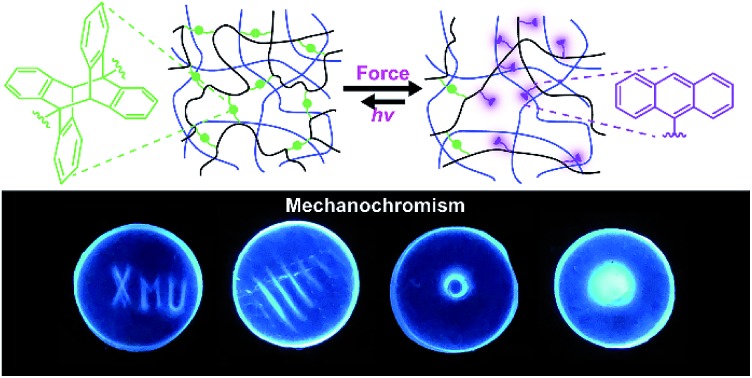
Multi-network elastomers with a reformable sacrificial network containing mechanochemically sensitive anthracene-dimer cross-links manifest reversible mechanochromism and remodeling.

## Introduction

Soft polymer networks, such as gels and elastomers, are important load-bearing materials thanks to their reversible deformability, which makes them well suited for diverse applications, including in emerging fields of wearable electronics,^1^ soft robotics,^2^ and tissue engineering.^3^ Polymer networks made of covalent cross-links are either stiff or tough but not both because their threshold fracture toughness is inversely proportional to the square root of the Young's modulus.^4^ Incorporating sacrificial bonds removes this correlation. Considerable success has been achieved with hydrogels incorporating both covalent and non-covalent cross-links.^5^–^7^ A complementary approach applicable to hydrophobic and solvent-free materials results in strong and tough double- and multi-network (DN or MN, respectively) hydrogels^8^ and elastomers.^9^ These materials are composed of a highly cross-linked pre-stretched stiff network,^10^ interpenetrated by one or more loosely cross-linked soft networks. The accepted toughening mechanism postulates that the stiff network bears most of the load and progressively ruptures, dissipating strain energy, whereas the soft network(s) prevents the formation of macroscopic cracks, keeping the integrity of the material.^11^

Loading an existing covalent multi-network material permanently elongates and softens it because the rupture of the stiff network is irreversible. The high degree of prestretching of the stiff network means that when it ruptures in a loaded sample, it locally relaxes, making the remaining network less effective at dissipating strain energy.^8^ Whether the original mechanical properties of the material can only be restored by regenerating the stiff network to the original degree of prestretching and how to do so, remain to be understood. Likewise, little is known about how the mechanical properties of the material depend on the integrity of the stiff network, its degree of prestretching and the density of the backbone bonds comprising the network. The reason in part is the current lack of methods to quantify bond scission events or the means to reform these bonds, even at the cost of decreasing the degree of prestretching.

**Fig. 1:**
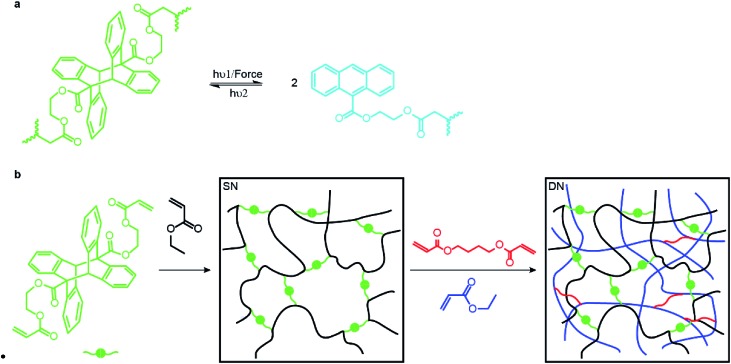
(a) The dissociation and dimerization reactions underlying this work. (b) Synthesis of multi-network elastomers. Ethyl acrylate is the monomer for all networks while anthracene dimer and butane-1,4-diyl diacrylate are the cross-linkers for the sacrificial and other networks, respectively.

Here we report the first step towards developing such methods. We describe the synthesis and proof-of-the-concept studies of the mechanical properties of double- and triple-network elastomers with the stiff (sacrificial) network containing anthracene-dimer mechanophores (Fig. 1a). Previously loading elastomers containing such anthracene dimers was demonstrated to produce emissive anthracenes,^12^ although the molecular mechanism of the dimer dissociation remains to be established.^13^ Thus, the reported MN elastomers containing anthracene dimers provide a means of monitoring the load-induced degradation of the sacrificial network. Because anthracenes photodimerize, the dimers are regenerated by irradiating partially degraded materials providing a means of testing how regeneration of dissociated bonds of the sacrificial network affect the mechanical properties of the elastomer.

## Results

### Preparation of multi-network elastomers

An anthracene dimer, bis(2-(acryloyloxy)ethyl) (5r,12r)-6-methyl-11-(*o*-tolyl)-11,12-dihydro-5,12-[1,2]benzenodibenzo[*a*,*e*][8]annulene-5,11(6*H*)-dicarboxylate, was synthesized from anthracene-9-carboxylic acid and 2-hydroxyetheyl acrylate in 3 steps and 55% overall isolated yield (see ESI†) as the head-to-tail isomer (Fig. 1b) because the alternative head-to-head isomer is thermally unstable.^14^ Photopolymerization of the anthracene dimer (green dots in Fig. 1b) with ethyl acrylate (black) in CH_2_Cl_2_ yielded a single network (SN) elastomer. Upon dialysis and drying it was swollen with EA (monomer), butane-1,4-diyl diacrylate (cross-linker: red short lines in Fig. 1b) and the UV initiator (not shown) and irradiated to yield a double-network (DN) elastomer, 17.5% of which by mass consisted of the stiff (dimer-containing) network. A triple-network (TN) elastomer was prepared from this DN elastomer by repeating the above procedure: in this elastomer, the stiff network accounted for 3.7% of mass. All elastomers were dried under vacuum at 80 °C to remove excess solvent after dialysis. We saw no evidence of anthracene dimer dissociating during photopolymerization, consistent with it being transparent to the wavelengths used to initiate photopolymerization.

### Mechanochromic properties

**Fig. 2:**
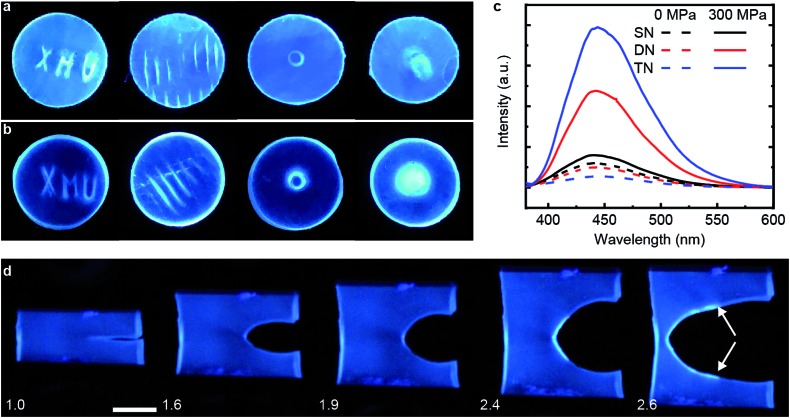
Mechanochromic responses of DN and TN elastomers. (a and b) Spatial localized mechanochromism induced by compression on surfaces of DN (a) and TN (b) films. (c) Plot of fluorescent intensity under 365 nm UV excitation for uncompressed (dashed lines) and uniaxially compressed samples (300 MPa for 5 min, solid lines). (d) Fluorescent images (365 nm UV excitation) of a TN elastomer during crack propagation. Numbers are the stretch ratio along the vertical direction. The two white arrows indicate the start of the newly-formed surface. The scale bar is 1.0 cm.

We demonstrated mechanochromism of DN and TN elastomers by measuring an increase of the fluorescent emission of the samples in 3 loading scenarios. In all experiments, the emission intensity remained unchanged for months after removal of the load. First, we pressed each sample with stamps bearing raised features corresponding to letters, lines, a ring and a circle. The fluorescence enhancement was confined to these features, making them distinguishable over the background (Fig. 2a and b). Second, each sample was compressed uniaxially at 300 MPa for 5 min, which increased the fluorescence emission intensity of the DN and TN elastomer, respectively, 4.8-fold and 14.4-fold (Fig. 2c) over as-prepared samples. In contrast, this loading increased fluorescence of the SN elastomer negligibly (black line). The emission spectra were similar to those of 9-carboxyanthracene (Fig. S2†), suggesting that compression caused dissociation of the anthracene dimer. Finally, we observed increased fluorescence in the vicinity of a propagating crack (white arrows, Fig. 2d). For example, when a sample of TN elastomer with a 1 cm-long notch was stretched perpendicular to the crack plane by the stretch ratio (the ratio of the sample length along the vertical direction to its starting value) larger than 1.9 to induce crack propagation, the newly created interface manifested much higher fluorescent intensity than the rest of the sample. At the stretch ratio below 1.9 the crack did not propagate and the fluorescence intensity remained unchanged. The DN elastomer manifested a qualitatively similar but less pronounced emission enhancement (Fig. S11†). The results are qualitatively similar to a previous demonstration of mechanoluminescence during crack propagation.^9^,^15^

In all loading geometries, we observed no statistically significant increases in fluorescence for the SN elastomer or a DN elastomer in which anthracene dimer was dissolved in the material instead of being a part of the cross-links (Fig. 2c and S9†). Both results suggest against localized transient heating being responsible for dimer dissociation in the loaded samples. This conclusion is supported by the negligible dissociation of the dimer upon heating its solution to 120 °C for 12 h, as evidenced by its NMR spectrum (Fig. S10†).^16^,^17^

The data above indicate that anthracene dimers in the backbone of the sacrificial network in our MN elastomers dissociate to fluorescent anthracene in loaded samples. This mechanochromism enables visible detection and mapping of mechanical stress and chain-scission events in MN elastomers with high spatio-temporal resolution.

### Mechanical properties and damage recovery

**Fig. 3:**
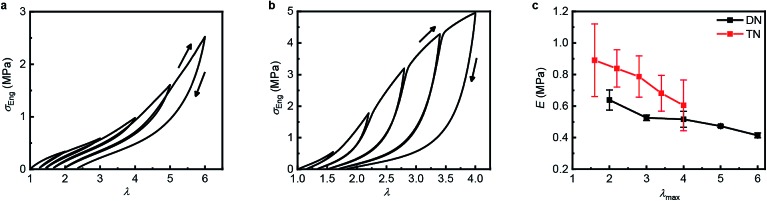
Step-cycle loading–unloading curves of multi-network elastomers. (a and b) Stress–stretch curves of DN (a) and TN (b) elastomers: *λ* was determined as the ratio of the separation of the two grips on the sample to its original separation. In each sequential stretching cycle, the sample was strained by 1.0 and 0.6 more than in the preceding cycle, for the DN and TN elastomers, respectively. (c) The Young's moduli as a function of the maximum stretch ratio *λ*_max_ of each loading cycle. Error bars are calculated from three independent tests and batches different from those used for Table 1 were used. Additional loading–unloading curves, demonstrating reproducibility, are shown in Fig. S14 and S15.†

Table 1 summarizes the key mechanical properties of pristine samples of the SN, DN, and TN elastomers and Fig. 3 illustrates the dependence of these properties on sample mechanohistory. The mechanical behavior is similar to that of other MN elastomers described in the literature.^18^ Increasing the number of sacrificial networks both stiffens and toughens the elastomer and increases the energy that is required for a crack tip to grow (critical energy release rate, *G*). Stretching an MN elastomer irreversibly damages the sacrificial network(s), with the extent of the damage increasing with the maximum stretch ratio, *λ*_max_, as evidenced by the increase in the stretch ratio at zero stress and in the onset of strain-hardening, and the decrease of the Young's modulus in each subsequent stretching cycle. The effects are larger for the TN elastomer compared to the DN analog. Mechanochromism and the evolution of the mechanical properties over multiple stretching cycles together suggest that the fragmentation of the sacrificial network(s) under loading occurs at least in part by dissociation of the covalently embedded anthracene dimers (Fig. 1a).

**Table 1:** Mechanical properties of the elastomers: the Young's modulus, *E*; fractional elongation at break, *λ*_b_; engineering stress at break, *σ*_b_, and the critical energy release rate, *G*_c_. Standard errors are calculated from three independent tensile tests. Typical stress–stretch curves are shown in Fig. S12 and S13

Entry	Mechanical Properties			
	*E* [MPa]	*λ*_b_	*σ*_b_ [MPa]	*G*_c_ [J m^–2^]
SN	0.28 ± 0.06	7.9 ± 0.9	1.0 ± 0.2	77 ± 5
DN	0.61 ± 0.07	7.2 ± 1.5	4.4 ± 1.6	155 ± 50
TN	0.88 ± 0.09	8.8 ± 1.3	7.0 ± 0.7	490 ± 70

**Fig. 4:**
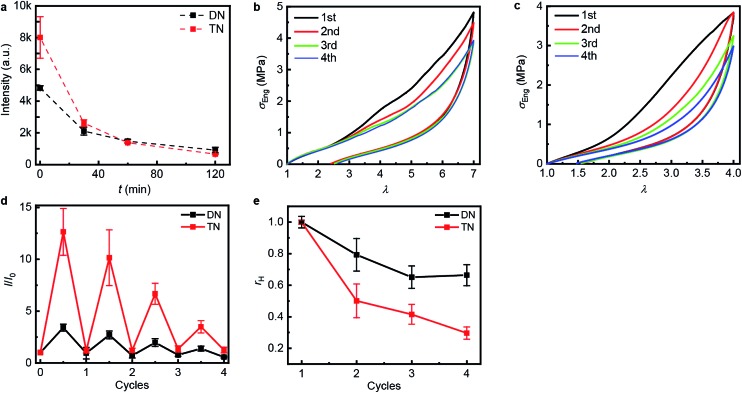
Optical remodeling of the DN and the TN elastomers. (a) The fluorescent intensity at 450 nm of the irradiated elastomers as a function of the irradiating time at 365 nm. (b and c) Stress–stretch curves of the DN (b) and TN (c) elastomer subject to sequential cycles of stretching-irradiation (1st: pristine, 2nd: after first irradiation, 3rd: after second irradiation, 4th: after third irradiation). (d) The ratio of the fluorescent intensity at 450 nm, *I*, at the maximum stretch ratio (half-integer cycle numbers) or after 2 h of irradiation (integer cycle numbers) to that of the same sample before stretching, *I*_0_, for sequential stretching-irradiation cycles. (e) The ratio of the area of the hysteresis curve of a sample, *A*, to that of the pristine sample, *A*_0_. Error bars in (d and e) are from three independent experiments. Additional stress–stretch curves are shown in Fig. S16 and S17.†

The propensity of anthracene to photodimerize potentially allows the sacrificial network(s) to reform upon irradiation, *i.e.*, these elastomers may be optically remodelable. To access this capacity, we first uniaxially stretched samples to *λ*_max_ of 7.0 over 120 s for DN and of 4.0 over 60 s for TN. After removing the load, the samples rested for 24 h at ambient temperature and then were irradiated under 365 nm UV light to dimerize the mechanically generated anthracenes. The emission intensity of the stretched samples decreased proportionally to the irradiation time during the first 120 min (Fig. 4a), when the intensity reached that of pristine samples, suggesting that all mechanically generated anthracenes had been photodimerized to non-emissive dimers (the contribution of anthracene bleaching to the decreased fluorescence was ruled out in control experiments described below). In all subsequent optical remodeling experiments, samples were irradiated for 120 min.

Careful removal of the photoinitiator and unreacted acrylates from our elastomers by dialysis and vacuum drying allows optical remodelling using the same wavelengths as photopolymerization without the risk of anthracene photodimerization being accompanying by photochemical reactions of any residual acrylate groups (*e.g.*, from partially reacted bisacrylate cross-linker), which could otherwise cross-link different networks. Because during photopolymerization only the photoinitiator is non-transparent to the irradiation wavelengths and during optical remodelling of mechanically damaged material only anthracene is, these two steps are functionally orthogonal.

To quantify how efficient *in situ* photodimerization of mechanically generated anthracene is in restoring the mechanical properties of the material, we subjected each polymer sample to four sequential stretching-irradiation cycles (Fig. 4b and c). We observed partial recovery of the mechanical properties of the DN elastomer during the consecutive stretching-irradiation cycles, as evidenced by the shift of the onset of strain-hardening to higher *λ* and a decrease in the area of the hysteresis loop (Fig. 4b and c). As a result, even after 3^rd^ stretching-irradiation cycles the DN elastomer retains 70% of its original energy dissipation capacity, a result comparable to that in state-of-the-art hydrogels containing both covalent and non-covalent cross-links.^5^ It seems quite plausible that the dissipation capacity of the DN elastomer (*r*_h_ in Fig. 4e) continues to decrease after the 3^rd^ cycle, but the increment is below the accuracy of our measurements.

Monitoring the emission intensity of the samples throughout the stretching-irradiation cycles suggest that fewer anthracene dimers dissociate in each subsequent stretching, because the intensity at the maximum stretch of each subsequent loading (Fig. 4d, half-integer cycle numbers) is lower than it was at the end of the previous loading. This reduction cannot be ascribed to incomplete photodimerization because the emission intensity of each sample after 2 h irradiation at 365 nm decreased to that of pristine samples (Fig. 4d, integer cycle numbers). Nor can it be attributed to anthracene photobleaching during irradiation because subjecting an unstretched DN or TN sample to 6 cycles of a 12 h irradiation at 254 nm to photodissociate the dimers, followed by a 2 h irradiation at 365 nm to reform them photochemically, demonstrated no dependence of the emission intensities at the end of either irradiation period (Fig. S21 and 22†) on the number of preceding cycles.

**Fig. 5:**
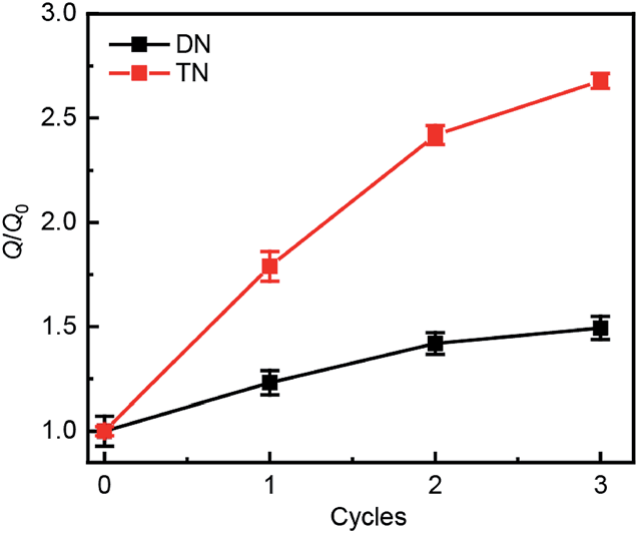
Normalized swelling ratio *Q*/*Q*_0_ of optically healed films. The samples were first subjected to an apparent compression stress of 300 MPa for 5 min before optical remodeling.

Swelling tests revealed that complete dimerization of mechanically generated anthracenes does not restore the original effective cross-linking density. Similar to the above remodeling test, DN/TN samples were first compressed at a nominal stress of 300 MPa for 5 min, rested for 24 h, and irradiated at 365 nm for 120 min. Irradiated samples were then immersed in THF and allowed to equilibrate. The degree of swelling, *Q*, was calculated as the ratio of the weight of the swollen sample to that before immersing it in THF. The normalized swelling ratio *Q*/*Q*_0_, (*Q*_0_, swelling ratio of pristine sample), is plotted in Fig. 5. For both DN and TN films, the observed increase in *Q*/*Q*_0_ with compression-healing cycles is consistent with either reduction in the cross-linking density or degradation of the non-sacrificial networks by scission of backbone bonds^3^,^13^ that are not restored by irradiation. Consistent with the data in Fig. 4b and c, mechanical activation seems more efficient in TN than DN and irradiation appears less efficient in restoring the first network of the TN elastomer compared to the DN analog.

## Discussion

Photodimerization of anthracene proceeds through a weakly bound excimer,^19^ which is formed either by a single-photon excitation of a molecular complex of two anthracene molecules or by association of an excited anthracene molecule with another anthracene. In either case, the modest association free energy limits how strained the two polymer strands linked by photochemically generated anthracene dimer can be. These strands are likely to be less strained that those formed by swelling of a network containing pre-formed dimers. As a result, photodimerization of all mechanochemically generated anthracenes, as is observed experimentally, probably requires rearrangement of the network to bring pairs of anthracenes in sufficient proximity to allow association and subsequent photodimerization with only modest distortion of the linked polymer strands from their minimum-energy conformations.

**Fig. 6:**
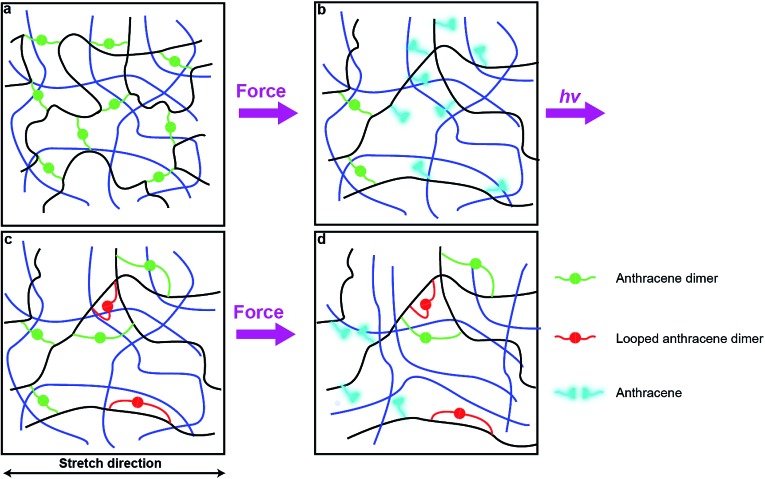
Proposed mechanism in remodeling and deformation cycles. (a) Anthracene dimers (green) in the sacrificial network (black lines) of an unstrained DN/MN elastomer. (b) Some dimers dissociate to fluorescent anthracenes. (c) Under 365 nm irradiation, anthracene moieties dimerize but some form ineffective cross-links like “loops” (red). (d) In successive loading, the load affects the most stretched chains strands of the sacrificial network the most, which contain fewer dimers than before loading/irradiation, resulting in lower fluorescent intensity. The cross-linkers in the second or third network (blue lines) are omitted for clarity.

One plausible consequence of this minimization of strain is to form loops connecting anthracenes bound to the same polymer chain (Fig. 6c). The accumulation of these regenerated but weakly-strained dimers in subsequent stretching-irradiation cycles may explain both the decreasing mechanochromic response of the material in subsequent loadings and the degradation of its mechanical properties (Fig. 6d). However, even strain-free loops may endow the material with some residual dissipation capacity as we demonstrated previously by single-molecule force experiments of polymer chains containing similar loops.^20^ If the length of the dimer-containing strap is significantly shorter than the contour length of the backbone that it constraints, stretching a chain containing such a loop strains the constraining strap and will cause mechanochemical dissociation of its dimer. Alternatively the dissipation may be due to some recoverable time-dependent behaviour due to an imperfect network structure containing pendant chains. These hypotheses await experimental validation.

## Conclusions

In summary, here we described the first example of covalent multi-network elastomers with reversible mechanochromic response to load, by virtue of their covalent cross-links of anthracene dimers. These materials complement the only other mechanochromic multi-network elastomers reported to date, containing a dioxetane derivative,^8^ in that the mechanochromic response of our materials is persistent rather than transient and the fluorogenic moiety (anthracene dimer) is regenerated upon irradiation of the material. In proof-of-concept experiments we demonstrated the utility of anthracene-dimer cross-linkers for monitoring mechanochemical remodeling of multi-network elastomers under cycling mechanical load. Stretching or compressing these elastomers made them fluorescent and irradiating them eliminated the fluorescence by regenerating anthracene dimers.

Complete photodimerization of mechanochemically generated anthracenes partially recovered the mechanical properties, with the area between the stretching and relaxation curves of the double-network elastomer remaining >70% of the original value even after 3^rd^ and 4^th^ loading/healing cycles. The same parameter for the triple-network elastomer was <40% and decreased faster with each subsequent loading/healing cycle. The results of the swelling tests were consistent with the stress–stretch curves. It remains to be established how much incomplete recovery of the mechanical properties of the elastomers results from the decrease in the strain of the photochemically regenerated sacrificial network and how much from mechanical degradation of the non-sacrificial network(s). Reformable mechanochromic cross-links, exemplified in this work by anthracene dimer, appear well suited for answering these and related questions about the molecular origin of the unique mechanical properties of multi-network elastomers.

## Conflicts of interest

There are no conflicts to declare.

## Supplementary Material

Supplementary information
