# Supplementary material for: Mechanochromism and optical remodeling of multi-network elastomers containing anthracene dimers
Source: Chem Sci. 2019 Jul 26;10(36):8367–73. doi: 10.1039/c9sc02580d (PMC6839589; doi:10.1039/c9sc02580d)
Supplement: Supplementary file 1 [file SC-010-C9SC02580D-s001.pdf]

Electronic Supplementary Material (ESI) for Chemical Science.
This journal is © The Royal Society of Chemistry 2019
Electronic Supplementary Material (ESI) for Chemical Science.

This journal is © The Royal Society of Chemistry 2019

Supplementary Information

Mechanochromism and optical remodeling of multi-network elastomers containing anthracene dimers

Huan Zhang[a], Dezhi Zeng[a], Yifei Pan[a], Yinjun Chen[c], Yonghong Ruan[a], Yuanze Xu[a], Roman Boulatov[b], Costantino Creton*[c], Wengui Weng*[a]*

[a] Department of Chemistry, College of Chemistry and Chemical Engineering, Xiamen University, Xiamen, Fujian 361005, P. R. China. E-mail: wgweng@xmu.edu.cn

[b] Department of Chemistry, University of Liverpool, Donnan Lab, G31, Crown Street, Liverpool, L69 7ZD GB, United Kingdom. E-mail: boulatov@liv.ac.uk

[c] Laboratoire Sciences et Ingénierie de la Matière Molle, ESPCI Paris, PSL University, Sorbonne Université, CNRS, F-75005 Paris, France. E-mail: costantino.creton@espci.fr

Supporting Information includes Figures S1-S22 and Tables S1 and S2

Experimental Procedures

I. General Procedures

2-Hydroxy-2-methylpropiophenone (HMP, 99 %), Ethyl acrylate (EA, 99 %), 1,4-Butanediol diacrylate (BDA, 99 %), Anthracene-9-carboxylic acid (95 %), 4-diMethylaMinopyridine, Propionyl chloride were purchased from Energy Chemical. 2-Hydroxyethyl acrylate (99 %) were obtained from Aladdin. Ethylene glycol (98 %) was obtained from TCI. THF was dried with Na before use. Dichloromethane were distilled over CaH_2 under nitrogen. All the other reagents were purchased from Sinopharm and used without further purification.

^{1}H NMR spectra were recorded in $CDCl_3$ (δ= 7.26 (^{1}H)) and referenced to the residual solvent signals on a 400 MHz Brucker AvanceII spectrometer at 25 °C. All chemical shifts were given in ppm (δ) as singlet (s), doublet (d), triplet (t), quartet (q), multiplet (m), or broad (br).

All the UV tests were performed in solution state on the SHIMADZU UV 2550. Fluorescence tests were performed in both solid and solution state on Hitachi F7000 with a PMT voltage of 400 V, unless otherwise stated.

Tensile test. Dumbbell-shaped specimens of SN, DN and TN were punched out of 0.7~2 mm thick SN, DN and TN films with a cutter. The gauge section was 2 mm wide and 5 mm long. Mechanical measurements were performed on an Instron 3343 instrument with the initial strain rate 0.05 s^{-1}.

Step-cycle test: The machine we used and the strain rate we set are the same as tensile test. And the stretch was determined as the ratio of the separation of the two grips on the sample to its original separation. In each sequential stretching cycle, the fresh DN sample was strained by 1.0 more than in the preceding sample. For TN the stretch ratio increase 0.6 in each cycle. The whole process lasted around 9 min and 5 min for DN and TN, respectively.

II. Small Molecule Synthesis

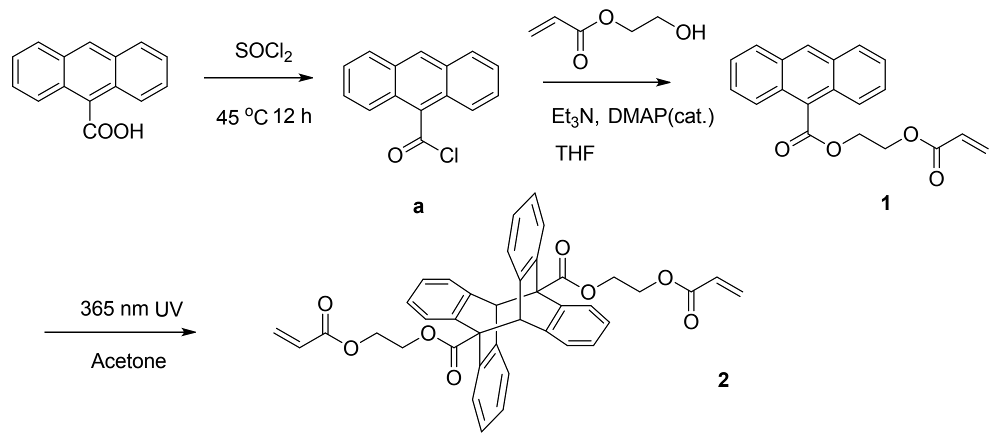

As the general synthetic route is shown above. The Anthracene-9-carboxylic acid was functionalized to achieve the anthracene-9-carbonyl chloride. Then, acrylic acid group was inserted to the end of the Anthracene-9-carboxylic acid to form the compound **1.** The anthracene dimer **2** was achieved from compound **1** under the 365 nm UV light for 12 h.

Synthesis of anthracene-9-carbonyl chloride (a)

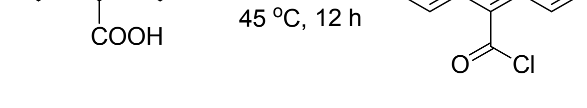

a

To a dry 100 mL round bottom flask, filled with sulfur dichloride (50 mL), anthracene-9-carboxylic acid (0.5 g, 2.27 mmol) was added. Then the stirred mixture refluxed at 45 °C for 12 h. After the solvent is removed, yellow powder

was achieved in 98 % yield. ^{1}H NMR (400 MHz, $CDCl_3$) δ (ppm) 8.59(s, 1H), 8.13 – 8.11(d, *J* = 8.99 Hz, 2H), 8.06 – 8.04(d, *J* = 8.90 Hz, 2H), 7.63(t, *J* = 7.34 Hz, 2H), 7.54(t, *J* = 7.59 Hz, 2H).

Synthesis of 2-(acryloyloxy)ethyl anthracene-9-carboxylate (1)

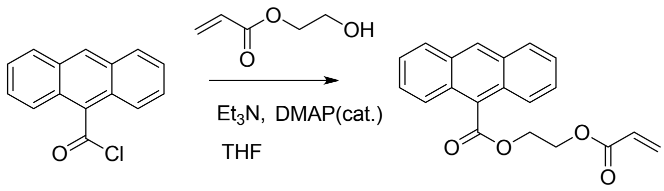

a **1**

Anthracene-9-carbonyl chloride (1 g, 4 mmol) which dissolved in 20 mL THF was added dropwise (in 30 min) to a cold stirred solution of 2-hydroxyethyl acrylate (10 mL, 95.3 mmol) and triethylamine (TEA) (0.55 mL, 4 mmol) in THF (30 mL) with dimethylaminopyridine (DMAP) (0.09 g, 0.78 mmol) as catalyst. The mixture was continually stirred at room temperature for 12 h. The organic layer was then washed sequentially with 1 M hydrochloric acid (HCl) (25 mL), 10 % sodium bicarbonate ($NaHCO_3$) (25 mL) and saturated brine (25 mL), and dried over anhydrous Na_2SO_4. The solvent was removed under vacuum, and the crude product was purified with column chromatography (CH_2Cl_2 / Hexane = 3 : 1) to give **1** in 90 % yield.^{1}H NMR (400 MHz, $CDCl_3$) δ (ppm) 8.55(s, 1H), 8.39 – 8.37(d, *J* = 8.54 Hz, 2H), 8.07 – 8.05(d, *J* = 8.54 Hz, 2H), 7.61(t, *J* = 7.87 Hz, 2H), 7.52(t, *J* = 7.87 Hz, 2H), 6.47 – 6.43(d, *J* = 17.08 Hz, 1H), 6.26(s, 2H), 6.19 – 6.12(t, *J* = 17.10 Hz, 1H), 5.84 – 5.82(d, *J* = 10.31 Hz, 1H).

Synthesis of bis(2-(acryloyloxy)ethyl) (5r,12r)-6-methyl-11-(o-tolyl)-11,12-dihydro-5,12-[1,2]benzenodibenzo[a,e][8]annulene-5,11(6H)-dicarboxylate (2)

A stirred solution of 2-(acryloyloxy)ethyl anthracene-9-carboxylate (**1**) (0.064 g, 0.2 mmol) in 100 mL acetone was sparged with N_2 for 15 min to remove the oxygen. The Pyrex was sealed with parafilm quickly and put under the 365 nm UV light for 24 h to get a white precipitate. The solvent and the remaining compound **1** were removed by filter. The white crude product was purified with column chromatography (CH_2Cl_2 / hexane = 2 : 1) to give **2** in 62 % yield. ^{1}H NMR (400 MHz, $CDCl_3$) δ (ppm) 7.01 – 7.00(d, *J* = 6.89 Hz, 4H), 6.87 – 6.78(t, *J* = 21.44 Hz, 8H), 6.69 – 6.67(d, *J* = 7.51 Hz, 4H), 6.43 – 6.39(d, *J* = 17.41 Hz, 2H), 6.14 – 6.07(t, *J* = 17.40 Hz, 2H), 5.88 – 5.86(d, *J* = 10.21 Hz, 2H), 5.73(s, 2H).

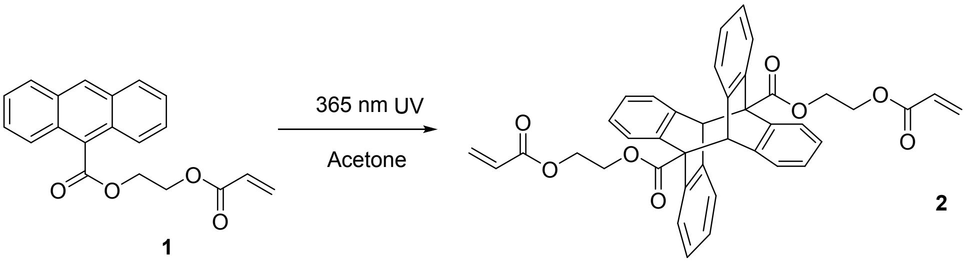

Synthesis of alkyl-terminated anthracene dimer (ATD)

ATD was prepared following the same route as cross-linker **2**, using typical organic synthesis method to form the compound **4**, the target ATD compound was obtained under the 365 nm UV light for 12 h.

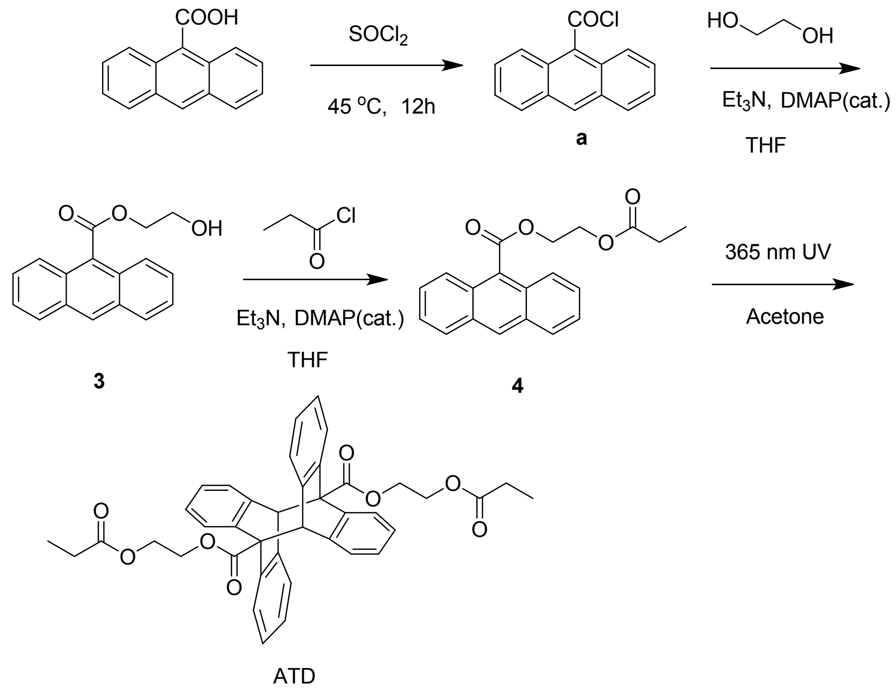

Synthesis of 2-hydroxyethyl anthracene-9-carboxylate (3)
Anthracene-9-carbonyl chloride (1 g, 4 mmol) which dissolved in 20 mL THF was added dropwise (in 30 min) to a cold stirred solution of glycol (2.23 mL, 40 mmol) and triethylamine (TEA) (0.55 mL, 4 mmol) in THF (30 mL) with dimethylaminopyridine (DMAP) (0.09 g, 0.78 mmol) as catalyst. The mixture was continually stirred at room temperature for 12 h. The organic layer was then washed sequentially with 1 M hydrochloric acid (HCl) (25 mL), 10 % sodium bicarbonate ($NaHCO_3$) (25 mL) and saturated brine (25 mL), and dried over anhydrous Na_2SO_4. The solvent was removed under vacuum, and the crude product was purified with column chromatography (CH_2Cl_2 / Hexane = 5 : 1) to give **3** in 95 % yield.^{1}H NMR (400 MHz, $CDCl_3$) δ (ppm) 8.56(s, 1H), 8.10 – 8.08(d, *J* = 8.59 Hz, 2H), 8.06 – 8.04(d, *J* = 8.45 Hz, 2H), 7.59 – 7.55(t, *J* = 8.07 Hz, 2H), 7.54 – 7.50(t, *J* = 7.07 Hz, 2H), 4.76(t, *J* = 4.54 Hz, 2H), 4.07(t, *J* = 4.06 Hz, 2H), 2.07(s, 1H).

Synthesis of 2-(propionyloxy)ethyl anthracene-9-carboxylate (4)
Propionyl chloride (1.93 g, 7.7 mmol) which dissolved in 30 mL THF was added dropwise (in 30 min) to a cold stirred solution of **3** (1.356 g, 5.1 mmol) and triethylamine (TEA) (1.1 mL, 7.7 mmol) in THF (40 mL) with dimethylaminopyridine (DMAP) (0.09 g, 0.78 mmol) as catalyst. The mixture was continually stirred at room temperature for 12 h. The organic layer was then washed sequentially with 1 M hydrochloric acid (HCl) (25 mL), 10 % sodium bicarbonate ($NaHCO_3$) (25 mL) and saturated brine (25 mL), and dried over anhydrous Na_2SO_4. The solvent was removed under vacuum, and the crude product was purified with column chromatography (CH_2Cl_2 / Hexane = 2 : 1) to give **4** in 88 % yield.^{1}H NMR (400 MHz, $CDCl_3$) δ (ppm) 8.52(s, 1H), 8.14 – 8.12(d, *J* = 8.71 Hz, 2H), 8.02 – 8.00(d, *J* = 8.36 Hz, 2H), 7.58 – 7.54(t, *J* = 8.42 Hz, 2H), 7.52 – 7.48(t, *J* = 7.14 Hz, 2H), 4.87(t, *J* = 4.59 Hz, 2H), 4.56(t, *J* = 4.37 Hz, 2H), 2.48 – 2.42(q, *J* = 15.10 Hz, 2H), 1.21(t, *J* = 7.41 Hz, 3H).

Synthesis of bis(2-(propionyloxy)ethyl) 5,12:6,11-bis([1,2]benzeno)dibenzo[a,e][8]annulene-5,11(6H,12H)-dicarboxylate (ATD)
A stirred solution of **4** (0.064 g, 0.2 mmol) in 100 mL acetone was sparged with N_2 for 15 min to remove the oxygen. The Pyrex was sealed with parafilm quickly and put it under the 365 nm UV light for 24 h to get a white precipitate. The solvent and the remaining compound **4** were removed by filter. The white crude product was purified with column chromatography (CH_2Cl_2 / hexane = 2 : 1) to give ATD in 73 % yield.^{1}H NMR (400 MHz, $CDCl_3$) δ (ppm) 7.01 – 6.99(d, *J* = 7.25 Hz, 4H), 6.86 – 6.81(m, 8H), 6.70 – 6.68(d, *J* = 7.39 Hz, 4H), 5.73(s, 2H), 4.63(t, *J* = 4.77 Hz, 4H), 4.35(t, *J* = 4.61 Hz, 4H), 2.35 – 2.29(q, *J* = 15.12 Hz, 4H), 1.13(t, *J* = 7.55 Hz, 6H).

III. Preparation of Multi-network Elastomers
Synthesis of single network SN
Single network SN was prepared by UV initiated free radical polymerization of a solution of EA as monomer, compound **2** as crosslinker and HMP as UV initiator. Prescribed amounts of reactants (EA, 0.9 mL, 8.46 mmol; **2**, 78 mg, 0.122 mmol; HMP, 0.1 μL, 6.74×10^{-4} mmol) were mixed into 3 mL chloroform and bubbled with nitrogen to remove any trace of oxygen, then the reactant solution in chloroform was injected into a 1mm thick glass mold, which

was then placed into a quartz flask under nitrogen atmosphere and exposed to the UV (Mercury lamp, 48 W). The polymerizations were left to proceed for 2 h. The sample was then extracted from the mold and immersed in dichloromethane for a week to extract any unreacted species. Dialysis bath was changed every day. Swollen SN was then dried under vacuum at 80 °C overnight. The resulting SN sample was then stored at room temperature for further use.

Synthesis of double network DN

A piece of the as-prepared SN was swollen in an oxygen-free bath composed of EA (21.27 mL, 200 mmol) as second monomer, BDA (3.8 μL, 0.02 mmol) as crosslinker and HMP (3 μL, 0.02 mmol) as UV initiator. Once swollen to equilibrium state, the sample was carefully extracted from the bath and placed in between two glass plates. The whole sample's holder was placed into a quartz flask under nitrogen atmosphere, which then exposed to the UV for 2 h to initiate and complete the polymerization. The sample was then extracted from the mold and immersed in dichloromethane for a week to extract any unreacted species. Dialysis bath was changed every day. Swollen DN was then dried under vacuum at 80 °C overnight. The resulting DN sample was then stored at room temperature for further use.

Synthesis of triple network TN

Triple network TN was prepared following the same process as DN but starting from a double network DN. The resulting TN sample was then stored at room temperature for further use.

To determine the composition of the final SN, DN and TN, we used the weight of the sample. The weight fraction (Φ^{1st}) of first network in the complete DN or TN was then calculated.

Synthesis of control single network ATD DN

Control ATD DN was synthesized by starting from a control single network SN with ATD dissolving in it.

Control single network ATD SN was prepared by UV initiated free radical polymerization of a solution of EA as monomer, BDA as crosslinker and HMP as UV initiator. Prescribed amounts of reactants (EA, 0.9 mL, 8.46 mmol; ATD, 78.5 mg, 0.122 mmol; BDA, 23μL 0.122 mmol; HMP, 0.1 μL, 6.74×10^{-4} mmol) were mixed into 3 mL chloroform and bubbled with nitrogen to remove any trace of oxygen, then the reactant solution in chloroform was injected into a 1mm thick glass mold, which was then placed into a quartz flask under nitrogen atmosphere and exposed to the UV (Mercury lamp, 48 W). The polymerizations were left to proceed for 2 h. ATD SN was directly dried under vacuum at 80 °C overnight for avoiding ATD running off from the film. The resulting sample was finally stored at room temperature for further use.

Synthesis of control double network ATD TN

A piece of the as-prepared ATD SN was swollen in an oxygen-free bath composed of EA (21.27 mL, 200 mmol) as second monomer, BDA (3.8 μL, 0.02 mmol) as crosslinker and HMP (3 μL, 0.02 mmol) as UV initiator. Once swollen to equilibrium state, the sample was carefully extracted from the bath and placed in between two glass plates. The whole sample's holder was placed into a quartz flask under nitrogen atmosphere, which then exposed to the UV for 2 h to initiate and complete the polymerization. The sample was then extracted from the mold and dried under vacuum at 80 °C overnight. The resulting ATD DN sample was then stored at room temperature for further use.

IV. ^{1}H NMR Spectra of All Compounds

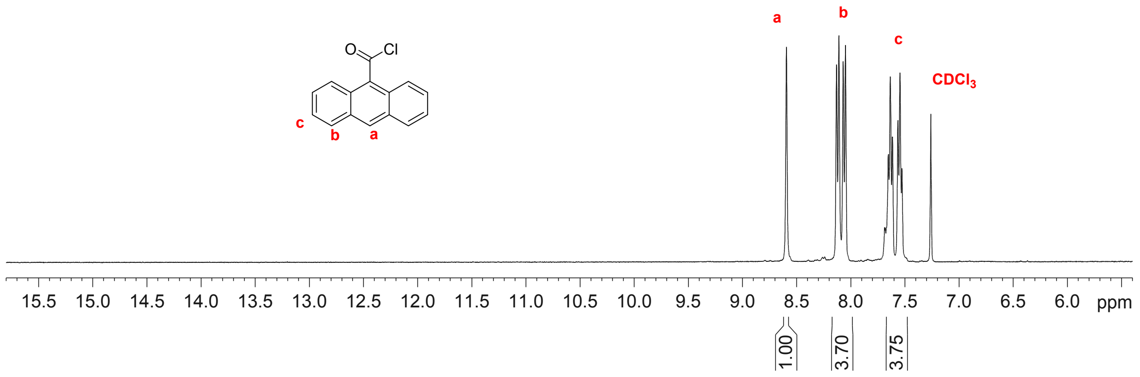

Figure S1. ^{1}H NMR (400 M) spectrum of compound **a** ($CDCl_3$, 7.26 ppm)

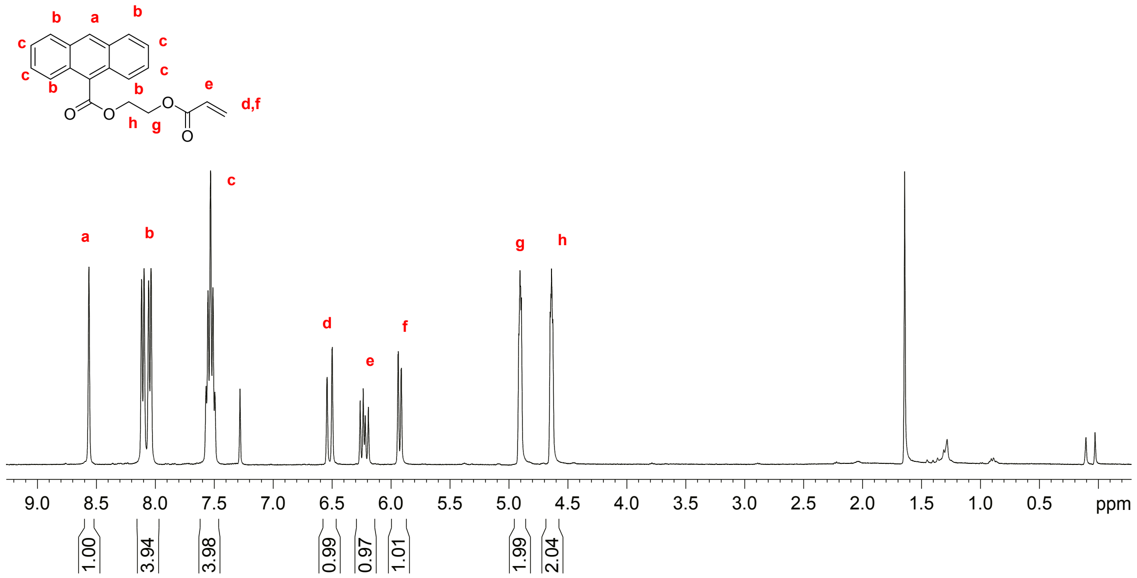

Figure S2. ^{1}H NMR (400 M) spectrum of compound **1** ($CDCl_3$, 7.26 ppm)

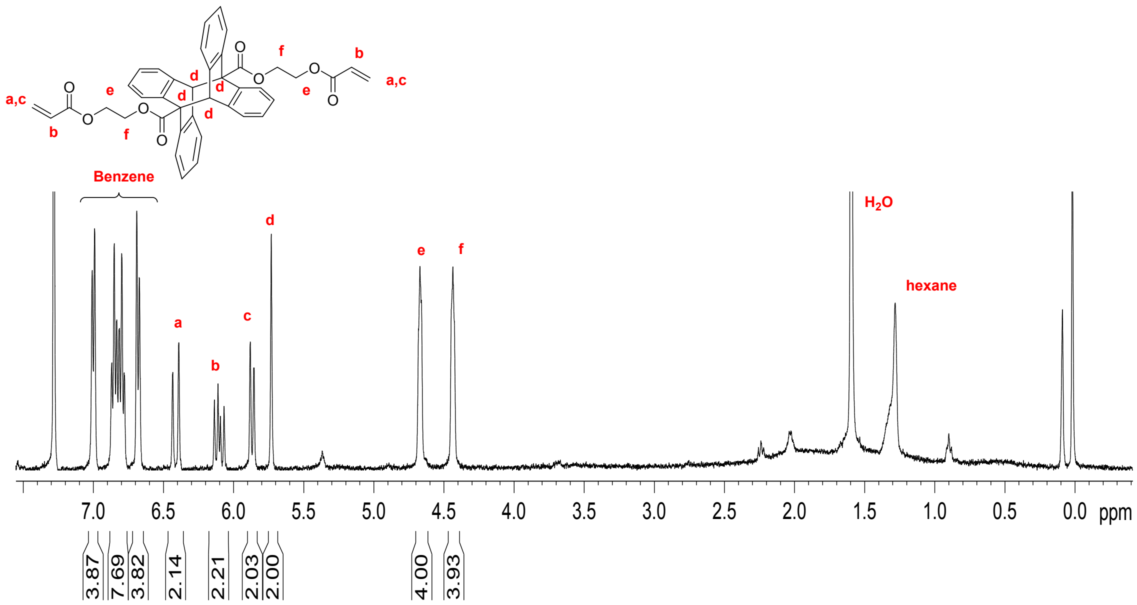

Figure S3. ^{1}H NMR (400 M) spectrum of compound **2** ($CDCl_3$, 7.26 ppm)

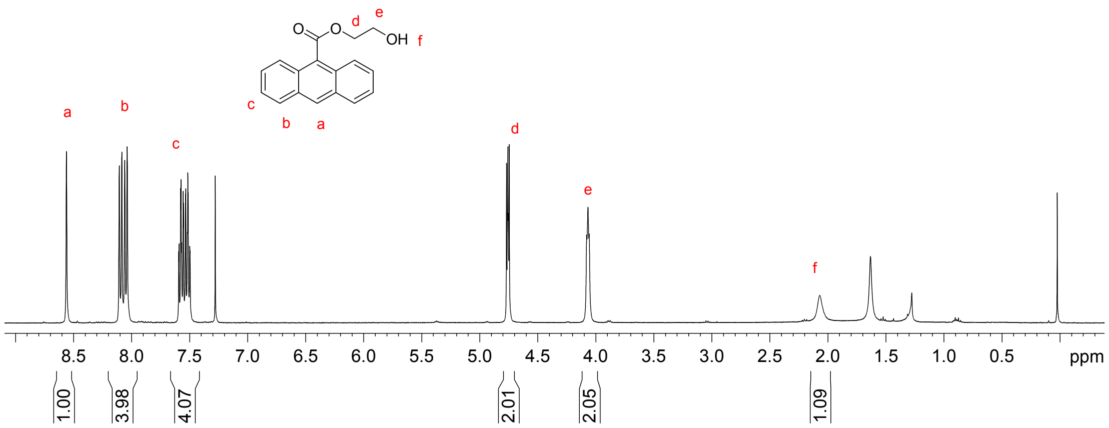

Figure S4. ^{1}H NMR (400 M) spectrum of compound **3** ($CDCl_3$, 7.26 ppm).

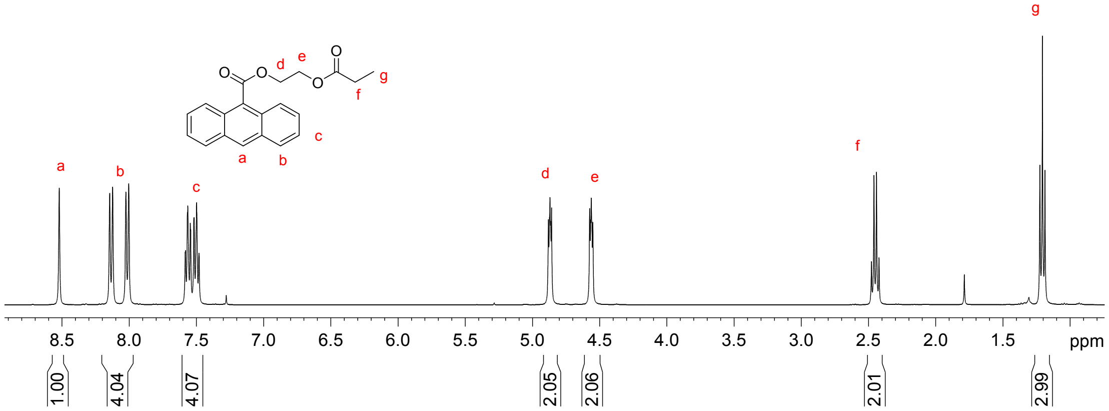

Figure S5. ^{1}H NMR (400 M) spectrum of compound **4** ($CDCl_3$, 7.26 ppm).

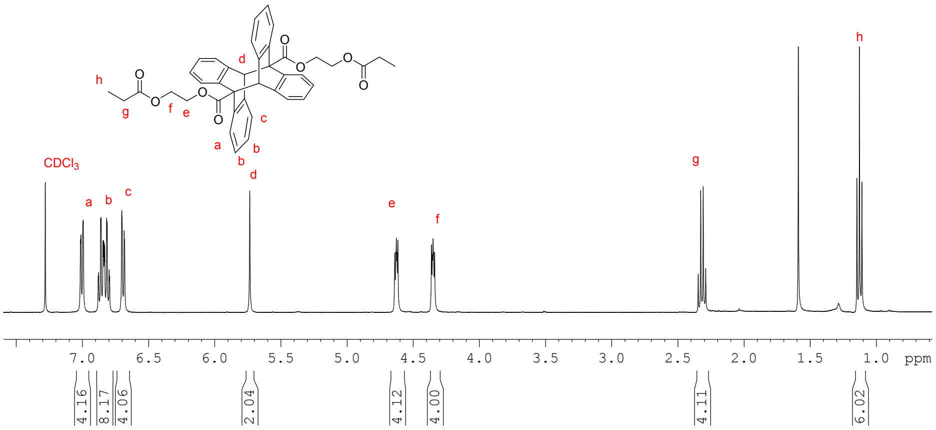

Figure S6. ^{1}H NMR (400 M) spectrum of ATD ($CDCl_3$, 7.26 ppm).

V. Results and Discussion

Table S1. The chemical composition of SN, DN, TN.

	Compound **2** (mmol)	BDA (mmol)	EA (mmol)	$CHCl_3$ (mL)	HMP (mmol)
SN	0.122	-	9.46	3	$6.74*10^{-4}$
DN	-	$3.89*10^{-3}$	43.49	-	$3.90*10^{-3}$
TN	-	$1.84*10^{-2}$	205.89	-	$1.85*10^{-2}$

Table S2. Weight fraction of first network Φ^{1st} wt % and concentration of anthracene dimer.

	SN	DN	TN
Φ^{1st} wt %	100 %	17.5 %	3.7 %
mol/L	0.135	0.024	0.001

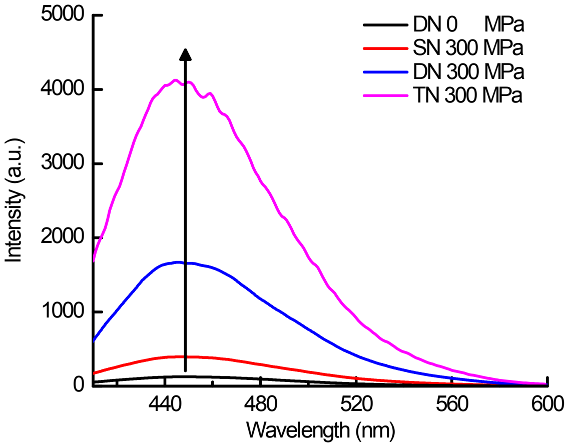

Figure S7. Fluorescent intensity of compressed SN, DN, TN samples at 450 nm.

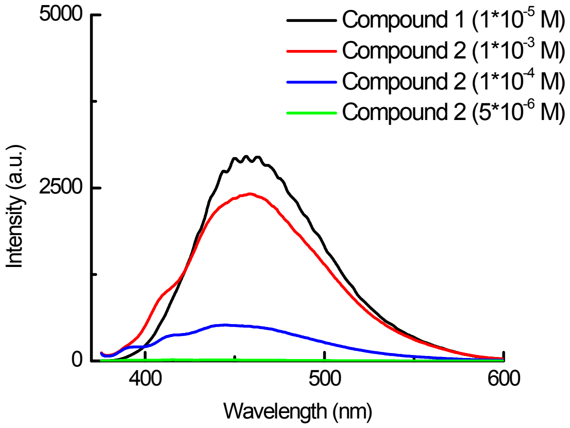

Figure S8. Fluorescence spectroscopy of compound **1**& **2** in $CHCl_3$ (the concentrations are shown in the figure).

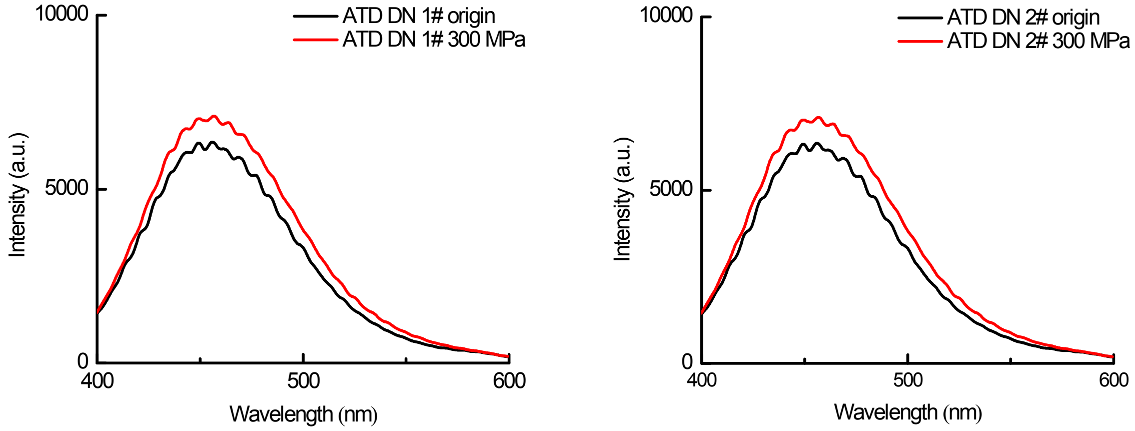

Figure S9. Fluorescent intensity of two compressed DN control samples with alkyl-terminated anthracene dimer (ATD) (0.024 mol/L) dissolved within the first network. The PMT voltage was 700 V.

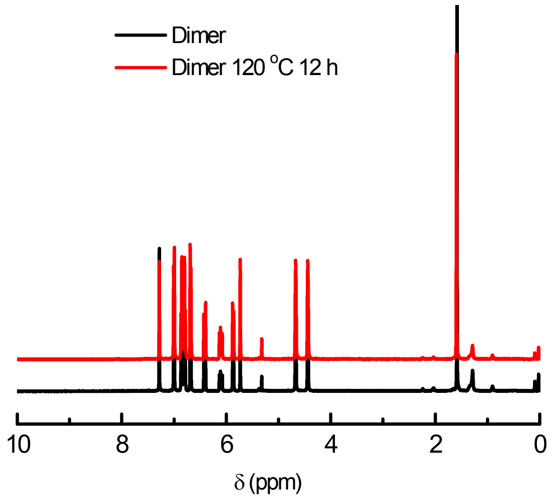

Figure S10. ¹H NMR of **2** heated at 120 °C overnight revealed no thermally induced decomposition.

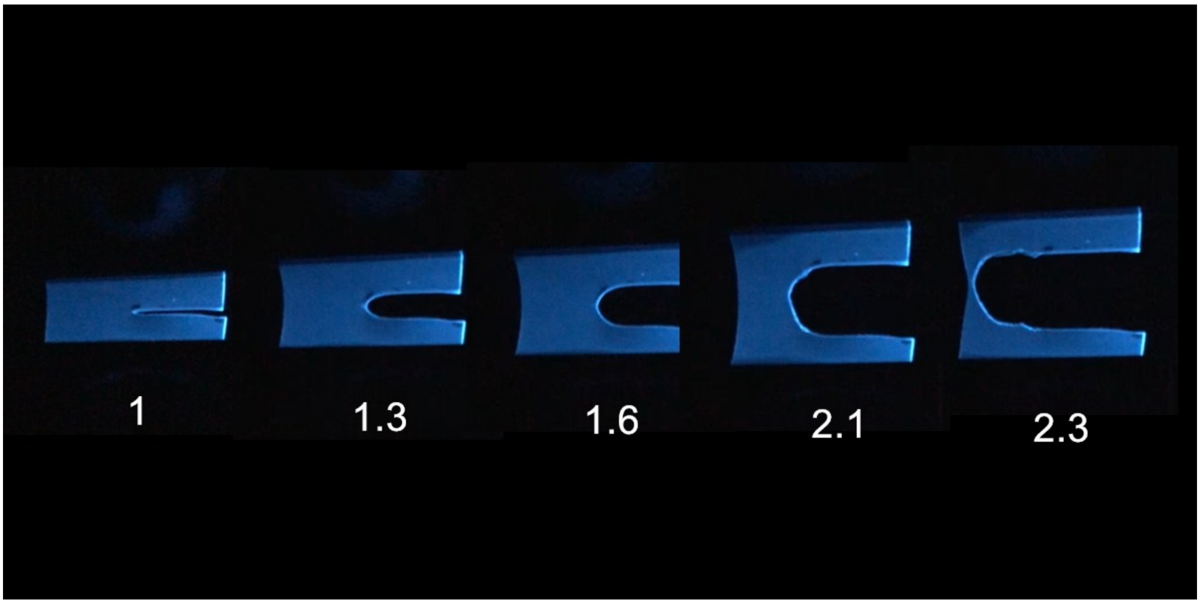

Figure S11. Fluorescent images (365 nm UV excitation) of a DN elastomer during crack propagation. Numbers are the stretch ratio along the vertical direction.

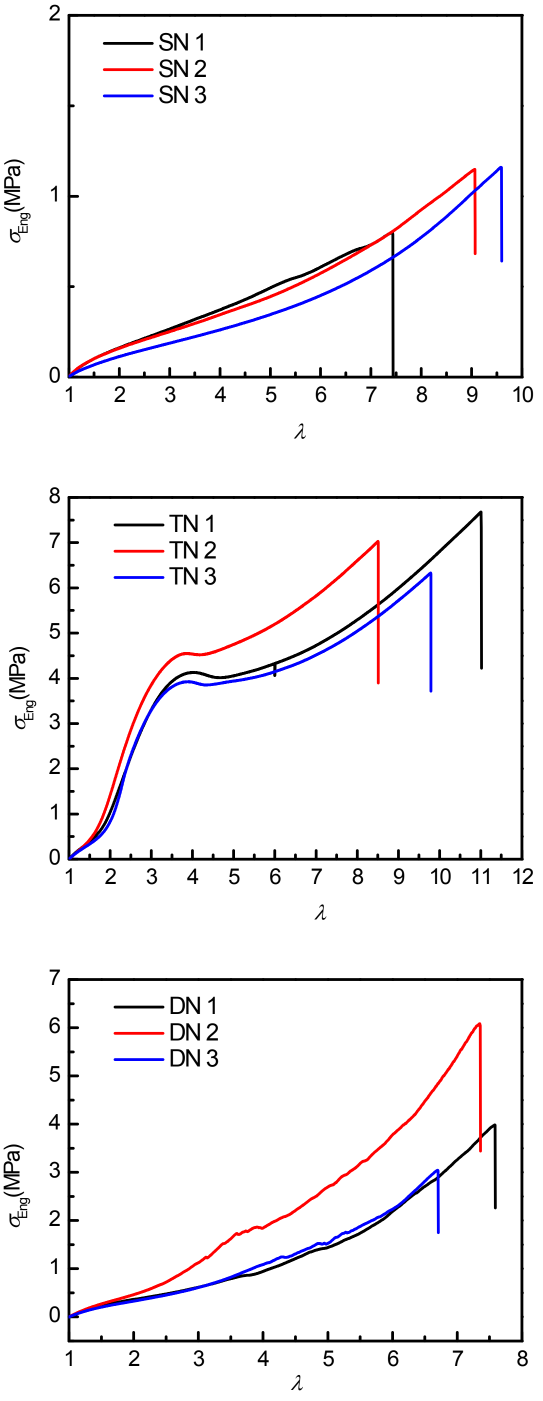

Figure S12. Stress-Stretch curves of SN, DN, and TN elastomers used for the calculation of mechanical properties in Table 1.

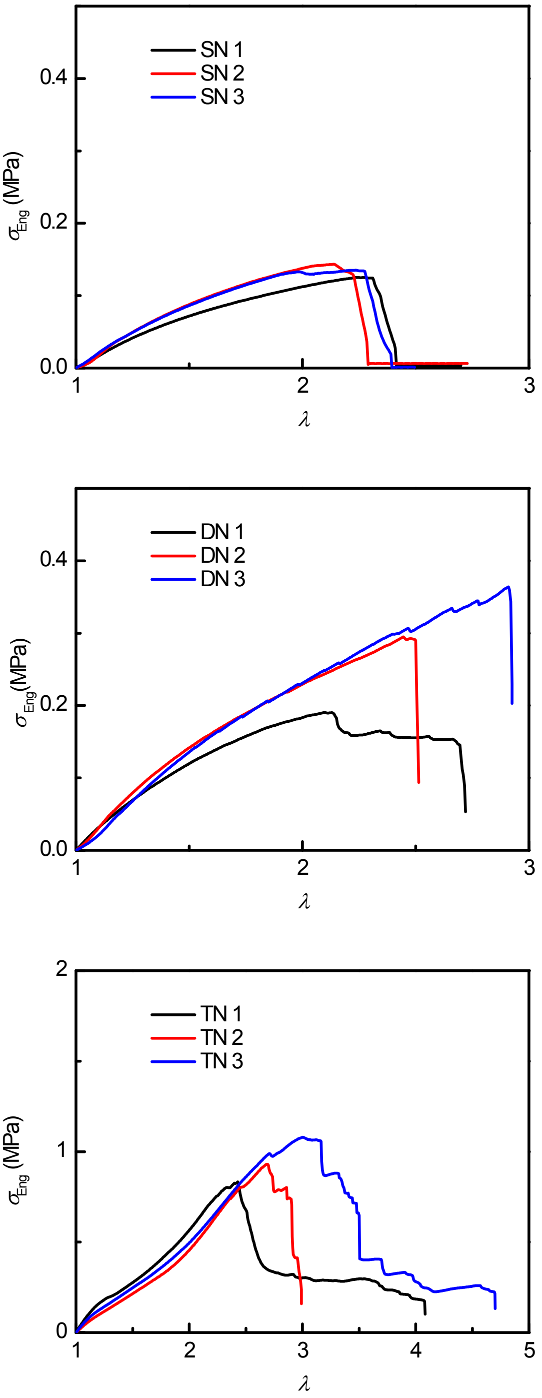

Figure S13. Crack propagation curves of SN, DN and TN elastomers used for the calculation of the critical energy release rate shown in Table 1.

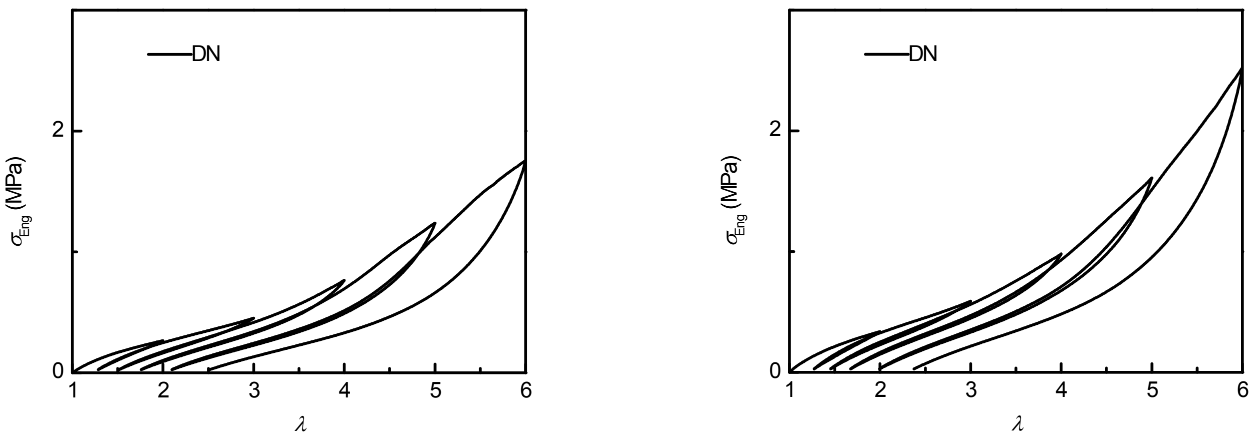

Figure S14. Additional step-cycle loading-unloading curves of DN elastomers.

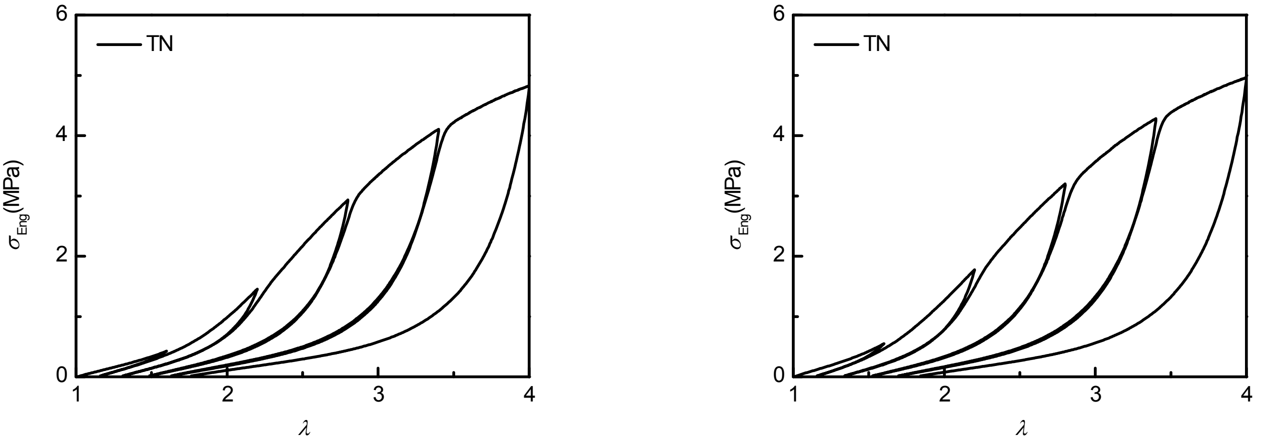

Figure S15. Additional step-cycle loading-unloading curves of TN elastomers.

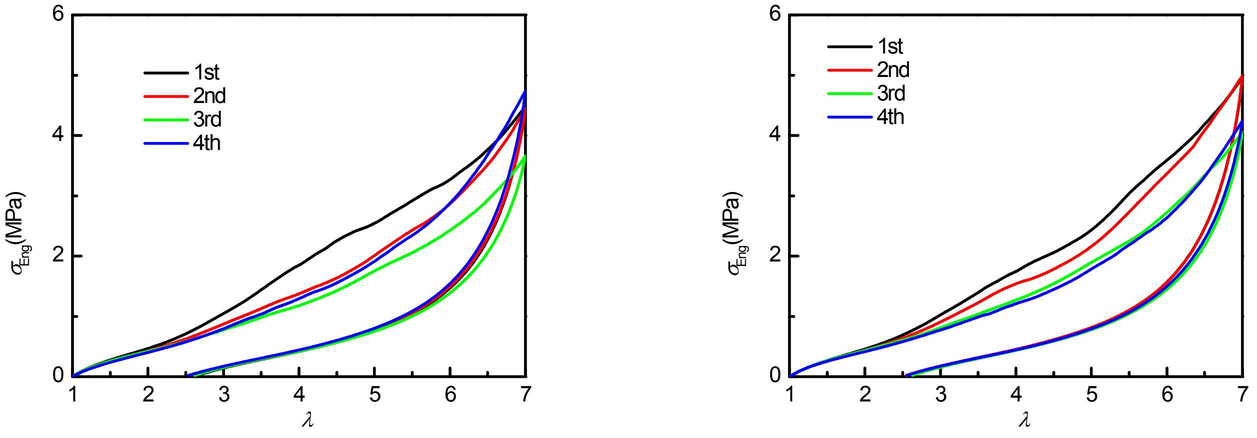

Figure S16. Additional stress-stretch curves of DN elastomers.

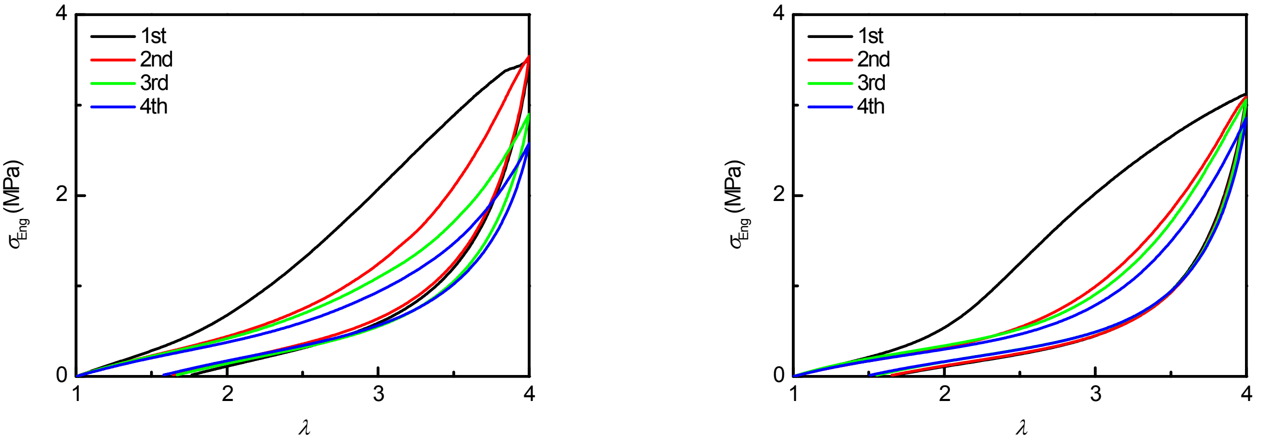

Figure S17. Additional stress-stretch curves of TN elastomers.

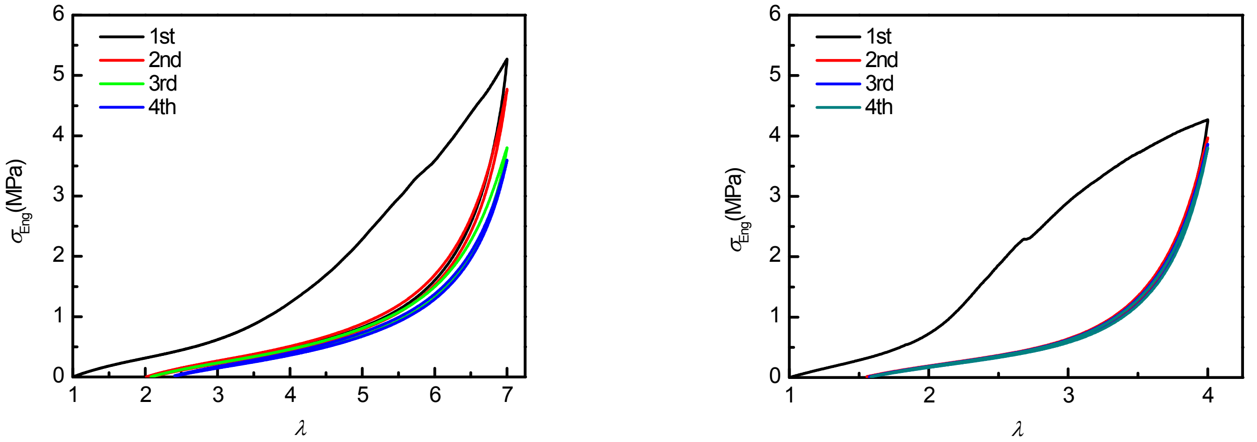

Figure S18. The consecutive loading-unloading loop tests without UV healing for DN (left) and TN (right) elastomers.

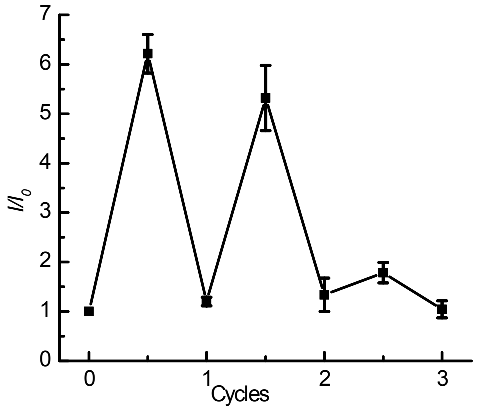

Figure S19. Fluorescence intensity of DN elastomer at 450 nm after compression and after healing.

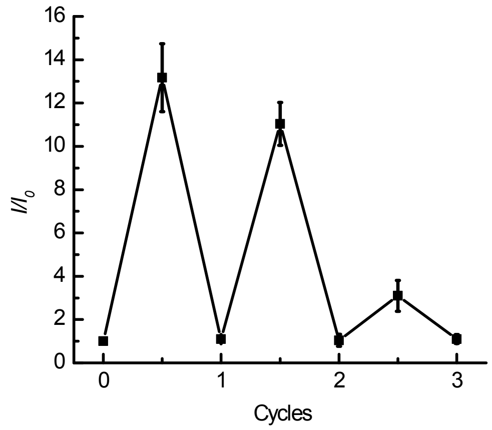

Figure S20. Fluorescence intensity of TN elastomer at 450 nm after compression and after healing.

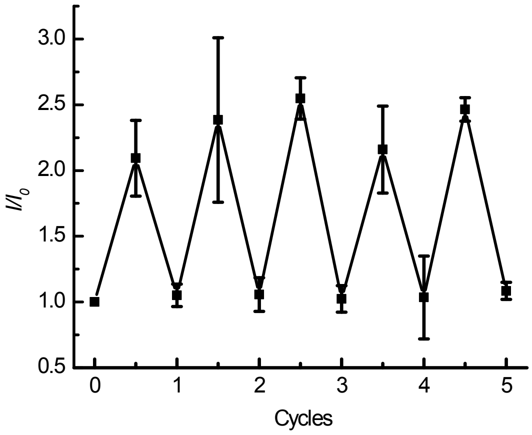

Figure S21. Normalized fluorescent intensity I/I_0 at 450 nm after UV irradiation (254 nm UV for 12 h, peak points) and after optical-healing (valley points, 365 nm UV for 2 h) of the DN elastomer as a function of cycles.

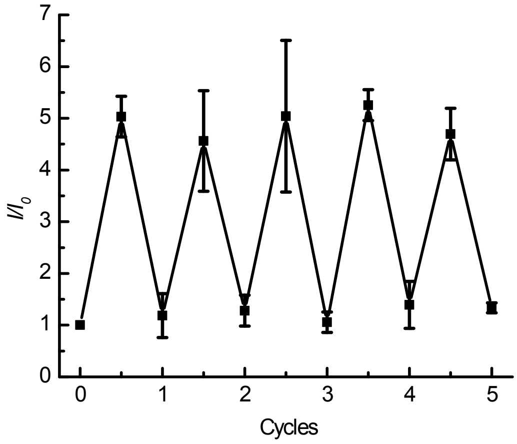

Figure S22. Normalized fluorescent intensity I/I_0 at 450 nm after UV irradiation (254 nm UV for 12 h, peak points) and afteroptical-healing (valley points, 365 nm UV for 2 h) of the TN elastomer as a function of cycles.

Author Contributions

W.W. initiated the idea and administrated the project. H.Z. co-supervised D.Z., Y.P., participated in experiment design and performed data analysis. D.Z. performed most of the experimental work. Y.P. assisted in synthesis. Y.C. assisted in fluorencence tests and supervised by C.C. R.B. and C.C. provided advices for tests and data analysis. H.Z., W.W.

and R.B. wrote the draft. Y.R. provided technical support. Y.X. participated in discussions. H.Z. and D.Z. contributed equally to this study.
